# Global Legal Environment for LGBTQ+ Sexuality and Public Health

**DOI:** 10.1017/jme.2025.60

**Published:** 2025

**Authors:** Matthew M. Kavanagh, Varsha Srivatsan, Florence Riako Anam, Ludo Bok, Luis Gil Abinader, Agrata Sharma, Catherine Grant, Yu Wei Chen, Sharonann Lynch

**Affiliations:** 1 Georgetown University, Washington, DC USA; 2 Center for Global Health Policy & Politics, O’Neill Institute for National and Global Health Law, Washington, DC USA; 3 Global Network of People Living With HIV, Nairobi, Kenya; 4 United Nations Development Programme, New York, USA

**Keywords:** LGBT health, human rights, discrimination, HIV

## Abstract

In 2023 the Supreme Court of Mauritius cited human rights and public health arguments to strike down a colonial-era law criminalizing consensual same-sex sex. The parliament of Singapore recently did the same through legislative means. Are these aberrations or a shifting global consensus? This article documents a remarkable shift international legal shift regarding LGBTQ+ sexuality. Analysis of laws from 194 countries across multiple years demonstrates a clear, ongoing trend toward decriminalization globally. Where most countries criminalized same-sex sexuality in the 1980s, now two-thirds of countries do not criminalize under law. Additionally, 28 criminalizing countries in 2024 demonstrate a de facto policy of non-enforcement, a milestone towards legal change that all of the countries that have fully decriminalized since 2017 have taken. This has important public health effects, with health law lessons for an era of multiple pandemics. But amidst this trend, the reverse is occurring in some countries, with a counter-trend toward deeper, harsher criminalization of LGBTQ+ sexuality. Case studies of Angola, Singapore, India, Botswana, Mauritius, Cook Islands, Gabon, and Antigua and Barbuda show many politically- and legally-viable pathways to decriminalization and highlight actors in the executive, legislative, and judicial arenas of government and civil society engaged in legal change.

## Introduction

Throughout history, LGBTQ+ people have faced varying levels of acceptance in national and international law. Differences in their legal treatment has varied across time, geographic location, and political regime.^1^ Studies of both history and social science show that same-sex intimacy has existed and flourished in diverse forms throughout the millennia and across the world’s cultures.^2^ But laws outlawing same-sex sexuality emerged and persisted for centuries in many cases, making regulation of LGBTQ+ sexualities a matter of state control — and one that has been inextricably tied up in forces of colonialism and geopolitics.^3^ It is simply impossible to separate the legal treatment of same-sex sexuality in a given country from transnational forces — and therefore it is most helpful to consider these laws through a comparative perspective.

These laws, meanwhile, have had significant impact on public health and are thus a question not only for the area of human rights and criminal law but also for health law. Law is a key factor shaping how states respond to people facing a pandemic — impacting who is at highest risk of infection and death.^4^ In the area of HIV, for example, a decade ago the landmark report from the Global Commission on HIV and the Law joined the AIDS movement’s calls for countries to remake their approach to consensual same-sex sex as an integral part of the response to the AIDS pandemic. “Rather than punishing consenting adults involved in same-sex activity, countries must offer such people access to effective HIV and health services and commodities,” they wrote.^5^ At the heart of this call was a recognition that states must choose whether to approach LGBTQ+ people as important participants in national response, in need of services and the protection of the state, or as people to be punished and criminalized. These two approaches stand in conflict. Even when deploying cutting-edge science, breakthrough biomedical tools, and a surge of services designed to reach gay men and other men who have sex with men (MSM), experience shows that law remains a critical factor. It can aid public health through protection or counteract it by increasing risk and driving people away from public health services.

This article sets out to document the trends on the criminalization of LGBTQ+ sexuality in recent years. In the first section, we review the context of criminalization of same-sex sexuality and the increasing move by normative bodies away from criminalization. In sections that follow, we describe how we set out to measure the criminalization of LGBTQ+ sexuality, including both a de facto and a de jure component, as well as legal protections against discrimination.

Some argue that we live in a “world divided” on LGBTQ+ sexuality.^6^ However, the “Contemporary Trends” section of this article shows a clear move worldwide toward abandoning criminalizing laws in most of the world. Exploring the period from 2018 to January 2024 shows more and more governments, at all income levels and in every geographic region, have decisively chosen to decriminalize consensual same-sex sexual acts under the law. Today, a clear majority of countries take a non-criminalizing approach — either because they never criminalized same-sex sex or because they have reformed their laws to decriminalize. Our research shows that many countries with criminalizing laws on the books have a de facto policy of nonenforcement. Together, these changes in law have had, and will have in years to come, important public health implications including on HIV, mental health, suicide, and beyond. The “Countertrends” section, however, shows that amidst this progress there is a clear effort to deepen criminalization in some countries — a transnational legal and political movement against a growing consensus. In “Health Impacts” we explore health effects of moving away from criminalization.

Finally, in “Legal and Political Pathways” we explore a set of case studies of law reform in some of the most recent countries that have decriminalized LGBTQ+ sexuality that illustrate how diverse champions from civil society and across government drive change through multiple legislative and judicial pathways to decriminalization. Together, this article shows an important global legal trend away from a criminalization approach to LGBTQ+ sexuality, with important public health benefits. But while this trend is clear, it is neither inevitable nor irreversible, and continued progress will require concerted effort and international action.

## Criminalization, Human Rights, and Public Health

Criminalization is both a legal categorization of certain acts and a sociopolitical process. As an empirical question about the state of the world, it reflects a combination of the simple existence of a specific statute that identifies some behavior as a violation of the law along with the actions of the state in enforcing and implementing a statute.^7^ These laws and their implementation are part of a broader social process through which state authorities, media, and public discourse define particular groups and practices as criminal, with a variety of negative consequences. Deciding to classify an act as “criminal” is, of course, just one of several approaches the state can take to regulating behavior^8^ — one “that leans heavily on threatened criminal penalties, criminal prosecution, and punishment.”^9^ Criminalization as such is more than simply the existence of a law imposing penalties for same-sex sex, but involves the way in which the law is enforced or implemented and how the people subject to the law are treated by the state and society. This creates significant variation in the degree to which people in different locations are subjected to the force of criminalization.

Criminalization has negative impacts for the criminalized that go beyond the specific punishment. It also has significant impact on the health of individuals and on public health — particularly well-recognized in HIV but with broader implications.^10^ A key *goal* of criminalization is stigmatizing actions or behavior deemed unacceptable by society. Yet in public health, we know that stigma is damaging to individual and public health. As Goldberg writes in the pages of this journal, “Because stigma has historically and continues to be so commonly inflicted on vulnerable and disadvantaged communities specifically by health professionals — even when they do not intend to do so — it is of great ethical concern.”^11^ Indeed, stigma is a major driver of health inequalities broadly, and especially in HIV.^12^

In addition, negative impacts include excluding groups from electoral politics, and “depriving them of material resources, social networks, family relationships, and legitimacy necessary for full political citizenship.”^13^ In the more recent period, criminalization has come to be seen as normatively harmful. This section outlines the history of laws outlawing same-sex sexuality and the way “criminalization” is constructed along with the growing consensus among both international human rights and public health institutions against criminalization.

### Criminalization of Same-Sex Sex

Most countries around the world have prohibited private, consensual same-sex sexual acts at some point.^14^ While a few countries like Vietnam and Cote D’Ivoire have never formally criminalized same-sex sexuality in the modern era, many, particularly those whose laws descend from Abrahamic faith legal strictures, made LGBTQ+ sexuality a subject for state regulation through criminalization and exclusion for hundreds of years.^15^ In the 13th and 14th centuries, as elite attitudes in Europe shifted sharply against same-sex intimacy, it was criminalized as “sodomy” on most of the continent — the “sin which men commit by having intercourse with each other, against nature and natural custom.”^16^ Sodomy was made a crime against the state under common law by Henry VIII in the 16th century.^17^

As European powers imposed colonial rule through much of the world, they brought legal codes on same-sex sexuality, many of which persisted long after formal colonialism.^18^ In what would become the US, many colonies copied English law, while Puritan leaders in colonies like the Massachusetts Bay and New Haven colonies promulgated even harsher penalties, decreeing death for men “lying with mankinde” and “unclean practices” or “carnall knowledge” between women.^19^ Indeed it is not a coincidence that India, Singapore, Malaysia, and other Asian former British colonies all have (or had) nearly identical provisions in Section 377 of their penal code. As one example, in Malaysia it reads:

*Carnal intercourse against the order of nature*

*377A. Any person who has sexual connection with another person by the introduction of the penis into the anus or mouth of the other person is said to commit carnal intercourse against the order of nature. Explanation — Penetration is sufficient to constitute the sexual connection necessary to the offence described in this section.*

*377B. Whoever voluntarily commits carnal intercourse against the order of nature shall be punished with imprisonment for a term which may extend to twenty years, and shall also be punished with whipping.^20^*

Some countries with Islamic traditions and some with French civil law have similar provisions, such as in Mauritania where the Penal Code of 1983 reads at Art 308:

*Any adult Muslim man who commits an immodest act or an act against nature with an individual of his sex will be punished by death by public stoning. If it is a question of two women, they will be punished as prescribed in article 306, first paragraph (3 months* — *2 years)^21^*

The laws persisted over long periods. At the start of the global AIDS pandemic in the 1980s — a beginning for our analysis — the vast majority of countries in the world criminalized same-sex sex.^22^ By our estimates, well over 120 countries and territories had laws making same-sex sex illegal.^23^

Criminalization of transgender people under these laws is complex.^24^ Trans women, for example, are often considered “men” under laws outlawing sodomy or same-sex sexuality and criminalized as such when they have sex with men.^25^ Yet even in countries where consensual same-sex sex is not criminalized, they can be subjected to state-sanctioned violence and exclusion. National legal environments range from deliberately denying the existence of transgender people to criminalizing transgender identities, to setting onerous preconditions on their ability to participate fully in society — or a mix of all three.

### Global Health and Human Rights Norms Converge Against Criminalization

In recent decades, both global public health and international human rights bodies have identified the removal of criminalization of LGBTQ+ people as aligned with science and international legal obligations.^26^ Law shapes how states respond to people facing a pandemic — what mix of support, coercion, and protection are offered, to which populations, and on what basis.^27^ Confronting a deadly virus, law impacts who is at highest risk of infection and death. The Global Commission on HIV and the Law over a decade ago called for a moratorium on enforcement and the full removal of laws against consensual same-sex sex.^28^ Criminalization, they argued, undermines efforts to end the AIDS pandemic.

This global norm shift has been a driver, one of several including growing movements and financial support for them, of the trends we document below against criminalization of LGBTQ+ sexuality. This is clearly seen in several of the case studies detailed below and help explain the trends we see.

In the 2021 Political Declaration on HIV and AIDS, United Nations (UN) member states committed to “ensuring that less than 10 percent of countries have restrictive legal and policy frameworks that lead to the denial or limitation of access to services by 2025.”^29^ The Global AIDS Strategy 2021–2026 adopted by the governing board (Programme Coordinating Board) of UNAIDS set out a plan with specific targets that less than 10% of countries criminalize same-sex sex.^30^ This article shows that important progress is being made on this aspect of the 10–10–10 targets set by UN member states, but that progress must be accelerated to achieve them.^31^ Today, the Global AIDS Strategy and the Global Commission on HIV and the Law have both provided broad articulation of norms against criminalization.^32^

Beyond HIV, the United Nations Development Programme (UNDP) and the World Health Organization (WHO) all recommend removing laws that criminalize consensual same-sex sex for broad public health impact.^33^

Not only does decriminalization of consensual same-sex sex have public health benefits, but authoritative interpretations of international human rights law show that criminalization of same-sex sexuality violates international legal obligations under several different principles.^34^ The UN Office of the High Commissioner has described how core international human rights treaties, including rights to non-discrimination, equal protection, life, privacy, and health, apply equally to LGBTQ+ people.^35^ UNDP has described this from a parliamentary perspective.^36^ The Yogyakarta Principles on the application of international human rights law in relation to sexual orientation and gender identity have provided an authoritative interpretation of how human rights apply across 29 principles in human rights from the right to life and rights to equality and non-discrimination to the right to effective remedies and redress.^37^

To start, discriminating on the basis of sexual orientation or gender expression and identity is a violation of human rights obligations. Articles 2(1) and 26 of the International Covenant on Civil and Political Rights (ICCPR) and Article 2(2) of the International Covenant on Economic, Social and Cultural Rights (ICESCR), among others, recognize the basic principles of non-discrimination and equal protection before the law.^38^ Sexual orientation and gender identity have also explicitly been recognized as internationally prohibited grounds of discrimination.^39^ The criminalization of same-sex sex clearly creates discriminatory and unequal protection for people of certain sexual orientations and gender identities, violating these human rights principles. In addition, as early as 1994, the Human Rights Committee found in Toonen v. Australia that consensual sexual activities in private fall within the scope of the right to privacy under Article 17 of the ICCPR.^40^ The right to privacy covers personal decisions and choices about consensual sexual relationships with others. Criminalizing same-sex sex arbitrarily and unlawfully interferes with this right.^41^

Applying the death penalty as a sanction against consensual same-sex sexual relations violates the right to life. The UN Human Rights Committee General Comment 36 states that “[u]nder no circumstances can the death penalty ever be applied as a sanction against” same-sex relations.^42^ As indicated in the Human Rights Committee General Comment 36, all laws and policies sanctioning same-sex sex with the death penalty violate the basic human right obligation to protect the right to life.

Article 12 of the ICESCR recognizes the “right of everyone to the enjoyment of the highest attainable standard of physical and mental health,” regardless of sexual orientation and gender identity.^43^ Criminalizing, discriminatory, and punitive laws and policies undermine health outcomes by instilling fear of being arrested or prosecuted and fueling stigma and social exclusion.^44^ Further, the lack of rights-affirming laws and policies curtails the LGBTQ+ community’s ability to access essential health services without discrimination; health information tailored to their needs, including sexuality and sex education; and key social services like employment, housing, education, and health care.^45^ As described in the “Contemporary Trends” section below, many of the courts and legislatures that have recently decriminalized have seized on both the public health and human rights arguments in their increasingly common decision to remove criminalizing laws.

These norms, of course, are not uncontested. As described in the “Counter Trends” section below, a concerted anti-rights movement has pushed back against the growing consensus in international human rights and public health. Indeed, Hadler and Symons argue world society is influenced by twin countervailing forces and that the best way to understand current politics on LGBTQ+ sexuality is as a “world society divided.”^46^ However true this may be, public opinion and social views are beyond the scope of this article. Our argument is that among international human rights and public health institutions, a clear consensus against criminalization has emerged and, as we describe below, this is increasingly mirrored in national laws around the world.

## Conceptual Framework for Measuring Criminalization

We set out to conduct an analysis of the relative criminalization faced by LGBTQ+ people in countries around the world. This has implications not only for the rights of those people, but also for public health since the legal environment in which programs to fight HIV and other diseases are run has implications for their effectiveness.^47^ This has long been theorized, but growing evidence, including that described in the “Health Impacts” section below, shows empirically that this is the case.

Since our goal is to measure the legal environment, we gather information and data to answer two key questions. First, the core question in this analysis is: Does national law/policy refrain from criminalizing and prosecuting people for consensual same-sex sexual acts? This question has two parts in the “Contemporary Trends” section below: we describe the de jure context of criminal law — whether a country has laws in place that make same-sex sex a crime. We frame this in the positive, asking whether a country refrains from criminalizing in written laws. In addition, conscious that some countries may have criminalizing laws on the books but have a policy of not enforcing those laws, we measure whether there is evidence that a country avoids prosecuting people for same-sex sexual activity. We label this as a de facto policy of nonenforcement, terminology we discuss below. For this indicator, since both are recommended by global norms, we label national laws for countries around the world as fully adopted if they have neither criminalizing law nor evidence of recent prosecutions; partially adopted if they have a criminalizing law but evidence of a non-prosecution policy under that law; and not adopted if people are criminalized both de jure and de facto for same-sex sex. Second, because simply not criminalizing is only one part of a pro-health, pro-rights legal environment, we also research a separate indicator on whether national laws include a ban on discrimination based on sexuality and gender identity.

### De Jure Non-Criminalization

Written law itself has important legal, social, and political effects. As Justice Kennedy of the US Supreme Court noted in the court’s ruling in Lawrence v. Texas, “When homosexual conduct is made criminal by the law of the State, that declaration in and of itself is an invitation to subject homosexual persons to discrimination both in the public and in the private spheres.”^48^ This is true even if the law is rarely enforced.^49^ In South Africa, for example, Edwin Cameron wrote how before decriminalization, rights of LGBTQ+ people were “not by right but by favour, by indulgence. We are dependent on, at best, the goodwill of the police to meet and act as we do and at worst are dependent on their blind eyes, their lack of knowledge or their inefficiency.”^50^ Since decriminalization of same-sex sexuality, LGBTQ+ South Africans still experience remarkably high rates of homophobic violence.^51^ But at the same time, research by Goodman found South Africans reported feeling relatively safe openly identifying as LGBTQ+ and felt able to go to the police for discrimination or harm perpetrated against them.^52^ Conversely, in other countries in the region where same-sex sex is criminalized, the opposite is largely true with strong feelings of unsafety that drive LGBTQ+ people away from services and few options for redress from violence, including that perpetuated by the police.^53^ This is particularly relevant in the context of HIV and populations of gay men and other men who have sex with men and transgender women, who globally face high rates of HIV.

For this indicator we ask the basic question: Does national law refrain from criminalizing consensual same-sex sexual acts? Information is collected from review of criminal statutes by the authors as well as data from the UNAIDS National Commitments and Policy Instrument (NCPI) and the International Lesbian, Gay, Bisexual, Trans and Intersex Association (ILGA) database.

### De Facto Policies of Non-Criminalization

Beyond the law as written, however, another important part of criminalization is whether and how the law is implemented or enforced. Given we are looking at all 194 countries around the world, we cannot possibly capture all the nuance and on-the-ground reality of how criminalizing laws are enforced. As an indicator, therefore, we measure whether a country’s policy is to avoid prosecuting people for same-sex sexual activity. We label this as a de facto policy of nonenforcement, using a concept and term widely used in literature on decriminalization^54^ and in studies of cross-national comparisons of law.^55^

Measuring prosecutions indicates the variation in decisions governments might make about when to use and who to subject to the force and punishment of the law.^56^ UN agencies have called for “establishing a moratorium on the application of laws that criminalize same-sex conduct between consenting adults.”^57^ Some countries have done so. President Festus Mogae of Botswana, for example, ordered the police in the early 2000s to never arrest people based on same-sex sexual conduct.^58^

To capture this, we researched the question: Has law-enforcement policy avoided prosecution for consensual same-sex acts in recent years? We use prosecutions within the last three years to indicate evidence of government intention rather than the actions of an individual officer or agent of the state that might occur in an arbitrary arrest.

## Methodology for Measuring Criminalization

The analysis in this paper draws on the methods of policy surveillance — the systematic, scientific collection and analysis of laws of public health significance over time^59^ — bringing it into a cross-national comparative context.^60^ A variety of qualitative methods including development of coding rules, comparative legal interpretation, policy analysis, and content analysis are deployed in constructing the dataset we used for the analysis below.^61^

Data on national policies below is generated as part of the HIV Policy Lab project^62^ via three approaches. First, we gathered a large number of primary sources (i.e., national laws and policy documents) through academic, civil society, and international organization networks, as well as internet searches. The text of laws and policies was coded using a “directed content analysis” approach and a dual-coder strategy with tests for intercoder reliability, using native speakers as the primary coder for the majority of texts.^63^ The text of all of the laws referenced in this report is available online in the HIV Policy Lab Resource Library at www.hivpolicylab.org/sources. Second, information formally submitted by governments to UNAIDS and the World Health Organization through the Global AIDS Monitoring (GAM) framework were also coded.^64^ Information from the GAM National Commitments and Policy Instrument questionnaire is shared publicly^65^ and includes information from governments every two years, with partial answers in interim years, and information from local civil society and non-governmental organizations on a set of complementary policy issues. This information is validated for internal consistency and completeness by UNAIDS and WHO — with illogical responses corrected, countries contacted in cases of missing data, and validation against primary sources for selected laws and policies. Third, a critical source of information for this analysis comes from ILGA World — the International Lesbian, Gay, Bisexual, Trans and Intersex Association, which maintains an extensive database of laws and policies.^66^ By collecting and coding data from multiple sources, it is possible to triangulate information and include the most up-to-date data available. Specifically, where we have multiple sources of information for the same country that conflicts, that triggers follow up research by senior legal analysts who consult primary sources and, where necessary, seek out national legal expertise to confirm correct interpretation. We did not find any cases where this deeper research was not conclusive. Our focus is on the content of the law and policy in a country and then on evidence of how the law has been enforced systematically. Policy as written has been described as a distinct “triangle” apart from implementation^67^ — separation that enables creation of comparable data and the opportunity to assess which may explain outcomes.

Data for the indicator on whether criminalizing laws are enforced or instead if there is a de facto policy of nonenforcement comes from primary and secondary research through content analysis of reports by human rights bodies, NGO reports, and credible news media as well as data from the NCPI and ILGA databases.^68^ We note that this data is necessarily imperfect and there is more uncertainty here than in other indicators, particularly in the category of non-prosecuting countries with criminalizing laws. To ensure validity, we have used only high-quality sources and wherever possible looked for corroborating information. In addition, we conducted validating interviews with local experts in over a dozen countries where we identified our sources were weakest. Nonetheless, there may well be cases where individuals have been, or are being, prosecuted that were not reported publicly and are not known to our interview subjects, and therefore not identified during our research.

This analysis advances the field of policy surveillance by providing one of the most robust applications of the method at a global level. Studying between-country differences, where most applications have focused on differences in law across subnational units, presents a particular challenge — as those who have undertaken this in other areas such as health security, abortion laws, drug laws, and beyond have found.^69^ We show not only a cross-sectional point-in-time surveillance, but an over-time longitudinal analysis — an ideal application of policy surveillance in certain ways. One lesson learned was that we often found different sources of information conflicted on the same country where policy change was in process, which took active further research to resolve. In addition, the exact data at which “the law” changed value (e.g. from not adopted to adopted) was not always straightforward. In legislative cases, for example, we had to decide whether to consider the date a law was passed or the date it went formally into force, noting that once the law had changed enforcement followed even if the “effective” date was later. On the other hand, *de facto* nonenforcement required us to allow for greater uncertainty as we depended not on legal text but on reporting. This specifically adds a greater chance of “Type II errors” — errors of omission or false negatives — in which we are unaware of actual enforcement. We deal with this methodologically as described above by triangulating multiple sources and conducting qualitative inquiry including interviews. But more important, it calls for a response through theory: we believe our results are a more valid measure of the phenomenon of interest (criminalization) if we include a nonenforcement measurement, and therefore we accept greater uncertainty.

Using this data, this article maps where national law refrains from criminalizing consensual same-sex sexual acts; where national law makes same-sex sex a crime but where there is no evidence of a policy of prosecuting under the law or, in at least one case, vice versa; and the most harmful criminalized legal environments, where same-sex sex is illegal under national law and there is evidence of a policy of prosecution under the law. Then it tracks an additional indicator on whether non-discrimination protections are included in law on the basis of both sexual orientation and gender identity. While these categories and the data behind them are imperfect, this gives the best available cross-nationally comparative view into the policy environment for LGBTQ+ rights and health.

## Contemporary Trends in Criminalization of LGBTQ+ Sexuality

### A Trend Toward Decriminalization Under Law

**Figure 1.**
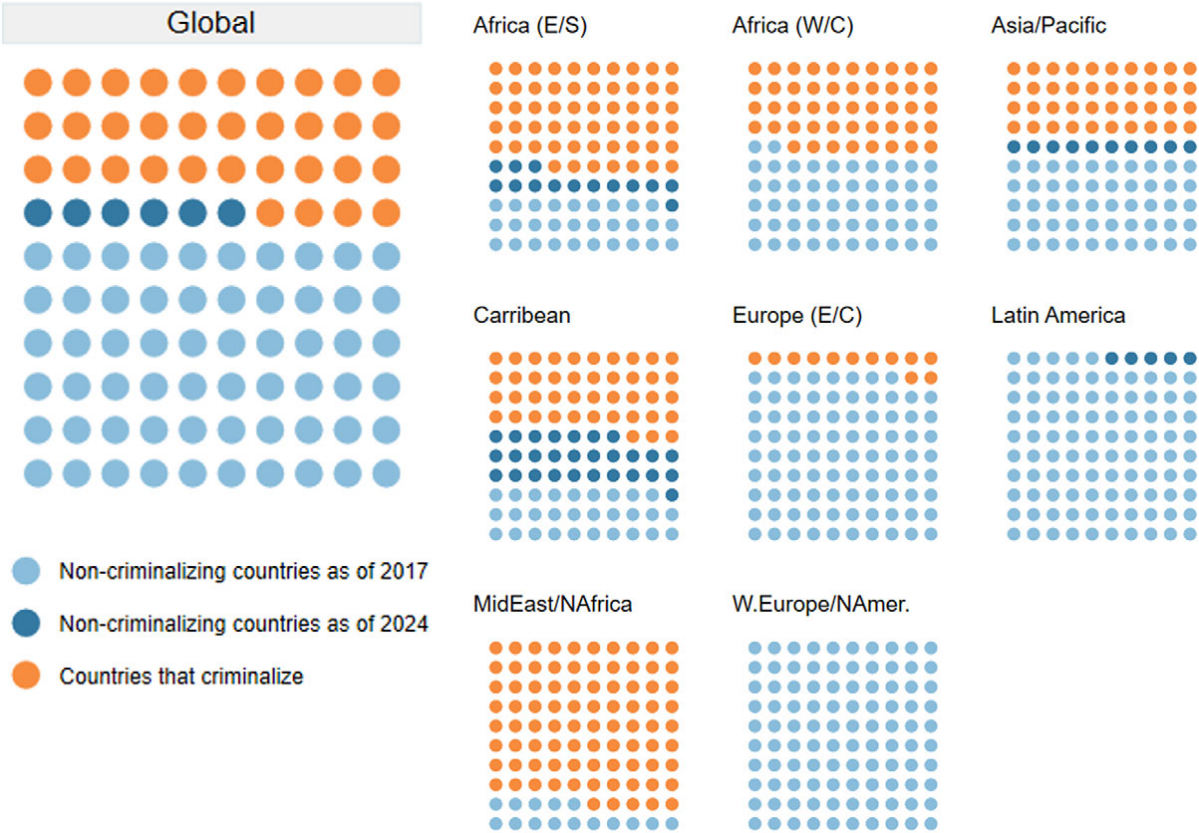
Progress of Decriminalization of Same-Sex Sexuality (2017, 2024)^74^

For the first time since the era of colonialism extended anti-sodomy laws across the globe, as of 2024 two-thirds of countries do not criminalize same-sex sexuality de jure. This is illustrated in Figure 1 below. As of January 2024, law in 129 countries (66% of the countries) avoids criminalizing consensual same-sex sex. There is significant regional variation in terms of the proportion of countries that do not criminalize. This includes nearly half (43%) of countries in Eastern and Southern Africa, more than half (52%) of countries in West and Central Africa, and 60% of countries in the Asia-Pacific region. Both in Latin America and the Caribbean and in Eastern Europe and Central Asia, more than 80% of countries have adopted non-criminalizing laws (82% and 87%, respectively), while all the countries in West and Central Europe and North America adopt non-criminalization. The Middle East and North African region show the lowest percentage of countries that do not criminalize same-sex sex. Bahrain, Djibouti, Israel, and Jordan are the only four of the region’s 20 countries where consensual same-sex sexual activity is not criminalized.^70^

Between 2017 and 2023, 13 countries have removed laws criminalizing consensual same-sex sexual activity.^71^ In 2022 alone, four countries — Singapore, Barbados, St. Kitts and Nevis, and Antigua and Barbuda — decriminalized consensual same-sex acts.^72^ In 2023, the Cook Islands and Mauritius joined them, and Venezuela removed its criminalizing military law.^73^ More countries decriminalized in 2022 than in any other single year of the past 25.

This trend represents a remarkable reversal in criminalization since the start of the AIDS pandemic. The HIV Policy Lab shows that in 2017, 117 countries did not criminalize same-sex sexual activity under the law, and by the start of 2024, that had risen to 129 — representing 66% of countries in the world.^75^ At the start of the AIDS pandemic, the figures were reversed — over 120 countries and territories had statutes criminalizing same-sex sexual activity.^76^ From another point, since the start of the global AIDS response and the resolution establishing UNAIDS in 1994, 46 countries have removed criminal laws.^77^

**Figure 2.**
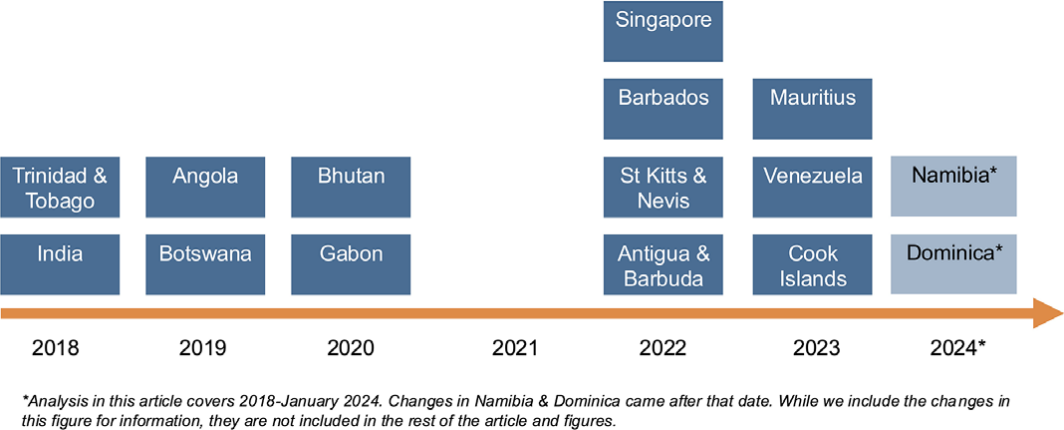
Decriminalization of Consensual Same-Sex Sex Under Law (2018-2024)^78^

Recently, countries from the regions of West and Central Africa, East and Southern Africa, Asia and the Pacific, Latin America, and the Caribbean and countries categorized as lower-middle, upper-middle, and high-income level have all decriminalized consensual same-sex sex. Since 2017, the fastest progress has been made in the Caribbean region, where the proportion of countries that criminalize same-sex sex in law has dropped from 71% in 2017 to 43% today. Figure 2 shows the countries that have decriminalized consensual same-sex sex since 2018.

In this period, one country, Gabon, newly criminalized in 2019 and then removed that law a year later.^79^ Indonesia introduced a new penal code that criminalizes sex outside of marriage, a law that will come into effect in 2026.^80^ As same-sex marriage is not recognized in Indonesia, this new law will effectively criminalize same-sex sex in all provinces in Indonesia in 2026 unless reversed before that time, where currently only one province specifically criminalizes same-sex sexuality.

This story of decriminalization in this period is also a story of decolonization. In its recent ruling, the Supreme Court of Mauritius pointed out, “Section 250 was not introduced into Mauritius to reflect any Mauritian values but was inherited as part of our colonial history from Britain. Its enactment was not the expression of domestic democratic will but was a course imposed on Mauritius and other colonies by British rule.” As described above, legal codes and laws criminalizing LGBTQ+ people and same-sex activity from Europe proliferated throughout the world under colonial rule. In the last decades, most of the countries that removed criminalization were removing colonial-era laws. Perhaps the most iconic of these is Section 377 in the Indian Penal Code 1860. Drafted during the British colonial era, it directly informed legislation throughout the British Empire and continue to inform criminalization provisions that exist in many countries. When the Indian Supreme Court finally struck it down in 2018, it marked an important turning point in global lawmaking.

Kenny and Patel note that countries that criminalize same-sex conduct are more likely to have been part of the former British Empire.^81^ Our analysis shows that out of 53 countries that belong to the Commonwealth or were formerly British colonies/territories, 38 of them criminalize same-sex conduct in 2023, most under colonial-era laws. About 34% of the world’s population, or 2.7 billion people, live in a former British colony or were part of the British Commonwealth, and yet these countries constitute more than half of the countries that criminalize same-sex conduct. This history belies the sense that criminalization of same-sex sex is “traditional” for much of the world.

Meanwhile, despite the wave of decriminalization, 65 countries continued to criminalize same-sex sexuality as of January 2024. As described in the “Counter Trends” section below, a number of these countries have moved to deepen criminalization and impose harsher penalties on LGBTQ+ people. Uganda and Brunei, for example, are among those that deepened criminalizing penalties.^82^ Among the countries that criminalize same-sex sex, Chad was the most recent country to enact laws newly criminalizing consensual same-sex sexual acts, in 2017.^83^ Significantly more work is needed to reach the United Nation’s 10–10–10 targets related to removing harmful laws. On the other hand, in response to significant international pressure, Brunei extended the moratorium on the death penalty to include cases under the new penal code showing progress is possible even amidst a harsh context.^84^

In addition to same-sex sexual activity, we note that there are only a handful of countries where transgender people are explicitly criminalized under a piece of legislation that makes “cross-dressing,” “impersonating the opposite sex,” or similar illegal. ILGA reported in 2020 that 13 countries had such laws;^85^ 20 countries reported to UNAIDS in 2023 that they criminalize transgender people.^86^ But there is clear evidence that in many more countries, public order offenses like indecency, vagrancy, and loitering are used systematically to subject transgender people to arrest and prosecution for their gender identity.^87^ Some countries are moving toward more human rights-based approaches, such as Spain’s legal reforms in February 2023, which allow for gender recognition procedures based on self-determination.^88^ In an important move, in 2022, Kuwait’s constitutional court struck down the law that criminalized “imitating the opposite sex,” which carried a prison term for cross-dressing.^89^ In 2018 the Caribbean Court of Justice, ruling in its status as appellate court for Guyana, struck down the vague cross-dressing laws that were used to harass and arrest transgender persons.^90^ In December 2020, the African Court on Human and People’s Rights issued an Advisory Opinion recommending that African states repeal or review vagrancy laws.^91^ They found that vagrancy laws discriminate against the most marginalized in society including transgender people, criminalizing the perceived status of an individual since they target “the poor and underprivileged, including but not limited to the homeless, disabled, gender-nonconforming, sex workers, hawkers, street vendors.” Since that time, courts in some African countries have found vagrancy laws to be unconstitutional.^92^ As steps toward more enabling jurisprudence from Africa, South Africa’s Act 49 and section 7B of Namibia’s Births, Marriages and Deaths Registration Act 81 of 1963 allow transgender persons who have begun gender transition to change their gender markers on their identity documents.^93^

### Partial Moves Toward Decriminalization Under De Facto Nonenforcement

One of the major recommendations of the Global Commission on HIV and the Law was placing an immediate moratorium on enforcement of laws criminalizing consensual same-sex sex, while advancing law reform.^94^ For the AIDS response, this is an important policy choice available to countries and can be observed whether they avoid prosecution under harmful laws. For example, in both Jamaica and Namibia, a de facto policy of nonenforcement has been important, albeit insufficient, for LGBTQ+ communities.

**Figure 3.**
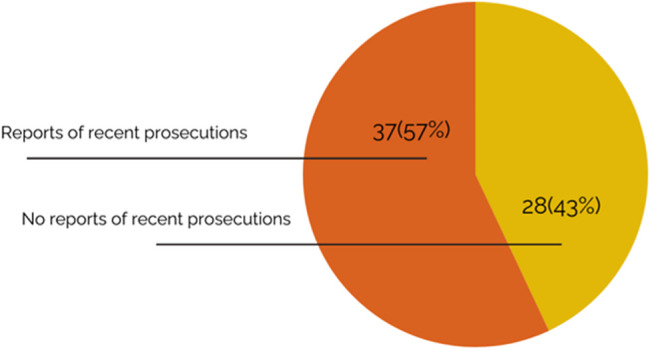
Evidence of Policy Enforcement of Criminalizing Laws (% of Countries with Criminalizing Laws), 2024

Twenty-eight of the 65 countries with criminalizing laws in 2024 appear to have a de facto policy of nonenforcement — with no reports of recent prosecutions identified in at least the last 3 years (See Figure 3). On the flipside, 37 countries did have reports of recent prosecutions of people for consensual same-sex sexual activity — evidence of a policy of active enforcement and criminalization of same-sex sexuality. The negative public health impact of criminalization is magnified where law and enforcement policies are combined — compounding stigma, discrimination, and fear that drives people away from health services.

Importantly, our analysis shows that every country that decriminalized same-sex sexuality between 2017 and 2024 previously had a de facto policy of nonenforcement. As of January 2024, according to the best available information, 28 countries that still have criminalizing laws did not have a recent history of prosecution, signaling this de facto policy. In 2017, this number was actually higher, at 35,^95^ because it was these countries that engaged in decriminalizing law reform. This shows that, as the Global Commission and others hoped, there is indeed an important pathway to law reform through moratoriums on enforcement.

### Summary of Global LGBTQ+ Criminalization

**Figure 4.**
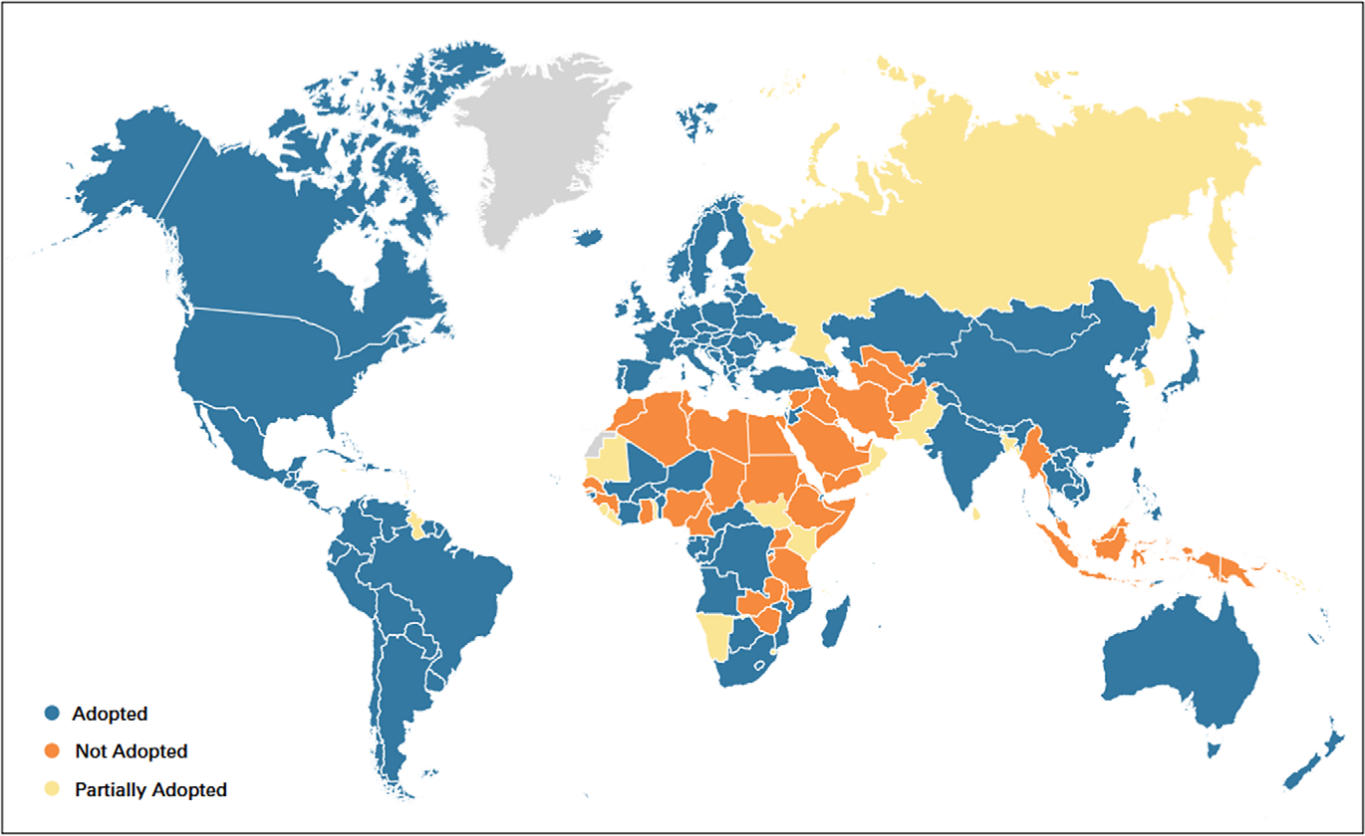
Criminalization of Same-sex Sex, January 2024.

Globally we can see a cascade of decriminalization if we combine these two indicators to classify countries on a three-part scale of adopted, partially adopted, and not adopted based on the questions described above. (Figure 4)

The most recent data available show that globally 66% (128/194) of the world’s countries have adopted a non-criminalizing approach to same-sex sex.^96^ This is remarkable and important, if incomplete, progress since the start of the AIDS pandemic.

Meanwhile, perhaps the most interesting group is the 28 countries that have partially adopted a decriminalizing approach, including those countries that have a de facto policy of nonenforcement.^98^ These countries may be well positioned for law reform in the near term — with policies that have accepted a less criminalizing approach but still have laws on the books that do not align with how the government is acting vis a vis LGBTQ+ people.

**Figure 5.**
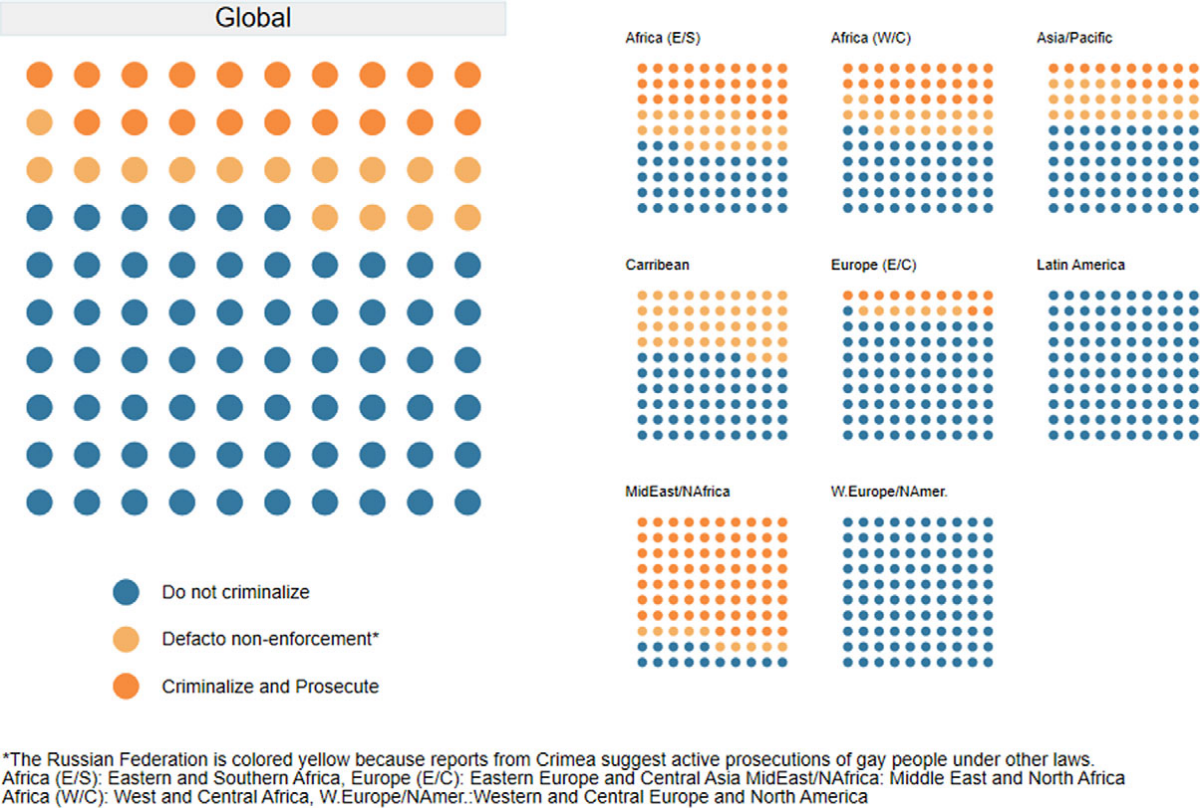
Progress toward Decriminalization of Same-Sex Sexuality (2024)^97^

If we combine the adopted and partially adopted countries, this number rises to 157 — close to 81% of the world. While the legal and/or law enforcement environment do not reflect the totality of experiences of LGBTQ+ populations, they form a strong foundation upon which a rights-based environment could be built. This also suggests the goal of less than 10% of countries with criminalizing laws is not out of reach, though countries currently actively criminalizing must shift (Figure 5).

But 37 countries (19% of the world) have not even partially adopted this policy — with the most harmful policy environment for public health of active criminalization with punitive laws combined with a policy of enforcement. As described in below, even amid this trend, progress has been met with coordinated counter efforts leading to further regression and entrenchment including persecution of LGBTQ+ communities in many countries, particularly where criminalization remains in place.

## Countertrends Toward Intensified Criminalization

A countervailing conservative international effort to redefine and constrain basic human rights has risen in parallel to the clear global move away from criminalizing LGBTQ+ sexuality.^99^ Challenging rights norms through a lens of nationalism and religion, this transnational movement has presented a challenge to liberal constitutionalism broadly, and especially on sexual and reproductive health and rights, LGBTQ+ rights, and the rights of religious and ethnic minorities.^100^ Our data as of January 2024 shows that this countertrend has not resulted in significant new criminalization — as shown in Figure 1 the trend is overwhelmingly toward fewer countries criminalizing. However, in multiple countries with criminalizing laws, there has been a push toward deepening criminalization and while we do not have quantitative data on this trend, it is clear on a qualitative level.

In some countries parliamentarians are actively seeking to intensify criminalization of same-sex sexuality rather than removing it. New laws and bills under consideration aggravate that criminalization by imposing harsher penalties, some including long prison sentences and even the death penalty for same-sex sexuality. Others are expanding the scope of criminalization, imposing sanctions on those who do not report others for same-sex sexuality or those who somehow support or assist affected populations, including gay men and other men who have sex with men, including as part of the AIDS response. And in those countries that do not criminalize, there is nonetheless efforts to criminalize non-sexual elements of LGBTQ+ life.

These laws are not just a product of national politics but what Joanna Kalb has called global “human rights proxy wars” wherein opposing sides from the United States and other wealthy countries move funding and political support to culture wars on different terrain.^101^

One example of these kinds of laws comes not in criminalizing gay sex specifically but couched as “anti-propaganda” laws. In the United States, for example, dozens of state laws limit the availability of LGBTQ+ related material in schools and libraries.^102^ Russia’s infamous legislation in this vein bans so-called propaganda of nontraditional sexual relationships.^103^ It represents “a synthesized and edited version of regional anti-propaganda laws that have been in effect in several districts (oblasts) in Russia since 2003.”^104^ The Constitutional Court, refusing to consider a complaint in 2010, wrote, “the family, motherhood and childhood in the traditional interpretation, received from our ancestors, are the values that provide a continuous change of generations, and are conditions for the preservation and development of the multinational people of the Russian Federation, and therefore require a special state protection.”^105^ These laws have since been replicated across the region.^106^ There is significant cross-national learning among politicians, such as from Hungary to the United States.^107^ Criminalizing speech regarding LGBTQ+ people, in and out of schools, criminalizes information and identities and disrupts advocacy for the health and rights of LGBTQ+ communities.

The second category of intensifying countries are those passing laws increasing criminal penalties for consensual same-sex sexual activities. Uganda has passed and implemented the most expansive recent law, increasing penalties for same-sex sexual conduct up to life in prison or the death penalty in cases deemed to be “aggravated,” while also criminalizing an extraordinarily broadly defined “promotion” of homosexuality (inclusive of acts as wide-ranging as renting an apartment to an LGBTQ+ person, being on the board of directors for an LGBTQ+ organization, or posting on social media in favor of LGBTQ+ rights) and creating a duty to report individuals suspected of being involved in homosexuality or other criminalized activities including the “promotion” of homosexuality.^108^ Iraq, Ghana, and several other countries have legislators drafting increasingly punitive laws.^109^ As of this writing, these bills are not law but have been accompanied by increased hate speech and targeted violence toward LGBTQ+ organizations and individuals. The bills deepening criminalization increasingly include the same sort of speech, advocacy, and “promotion” bans as the category above, in addition to increased penalties and reduced burdens of proof for same-sex sexual activity, as well as gender non-conforming dress.

Recent years have also seen a strong movement against greater protections for transgender people. As of this writing, almost 600 anti-trans bills have been introduced in US state legislatures in this year alone, according to the Trans Legislation Tracker.^110^ Many of these bills make using bathrooms consistent with one’s gender identity a crime or have criminalized gender-affirming care for young people or adults.^111^ In November 2022, Russia passed a law that banned references to and conversations about homosexuality and gender identity in public spaces.^112^ In May 2023, the Russian Parliament approved a bill outlawing gender-affirming care, which was then signed into law by the president.^113^ In the same month, Pakistan’s Federal Shariat Court struck down the 2018 protective legislation that enabled transgender persons to change their gender markers on official documents to match their gender identity;^114^ several appeals against this decision are pending.^115^ Hungary appears to have been one of the first countries in Europe to pass a law banning legal gender recognition.^116^

## Health Impacts of Non-Criminalization

It is increasingly clear in the field of HIV that removing criminalization and establishing a legal environment that is not punitive toward LGBTQ+ sexuality is beneficial to public health. As two authors recently wrote, “HIV is a story first written on the bodies of gay and bisexual men.”^117^ For example, around the world, gay men and other men who have sex with men, as well as transgender women, have higher rates of HIV than the general populations where they live.^118^ But the degree of risk varies based on their context. Gay men in Thailand have 12 times higher rates of HIV than all Thais, as expected. But in Malaysia the rates were 43 times higher than other adults at the last report — a difference that cannot be attributed to biology.^119^ One difference is that in Thailand same-sex sexuality has long been decriminalized, while Malaysia criminalizes under law — criminalization actively enforced. In this context, this article lays out an important basis for legal epidemiology — linking the close tracking of laws to health outcomes at the individual or group level.^120^ Beyond HIV, avoiding criminalization is associated with a variety of positive physical and mental health effects at the individual and public health level.

Decriminalization of consensual same-sex sex is an important factor in removing structural barriers driving HIV including stigma, discrimination, and socioeconomic factors that drive inequalities. Decriminalization opens pathways for engaging with public health and HIV services and facilitates an effective AIDS response — building trust among marginalized groups, enabling social movements and community-led responses, and reducing stigma, all of which open pathways to HIV testing, treatment, and prevention.^121^ Criminalization, by contrast, drives inequality and hinders progress toward achieving HIV epidemic control. Comparative analysis of outcomes in countries with and without criminalization reinforces our understanding of the public health benefits of removing laws criminalizing same-sex sexual activity.

Using the data on the legal status of LGBTQ+ sexuality that we outline here from the HIV Policy Lab, an analysis with some of the authors of this article and epidemiologist colleagues, led by Carrie Lyons, compared the situation of gay men and other men who have sex with men in African countries with varying degrees of criminalization.^122^ It combined the results of a set of individual-level studies of HIV rates that have been conducted over time and classified them according to the legal environment. That analysis showed those living in countries with laws that de jure do not criminalize same-sex sexuality had five times lower odds of living with HIV compared with men living in settings with criminalizing laws. Criminalization is associated with higher HIV rates. In addition, that analysis showed that gay men and other men who have sex with men living in settings where criminalizing laws were enforced with recent prosecutions had 12 times the odds of living with HIV than individuals in settings without recent prosecutions. Not only are HIV rates lower in non-criminalized contexts, but the disparity between gay men and other men is smaller where they do not face criminalization.^123^ In non-criminalized countries there was a 7.2 percentage point difference in HIV prevalence between MSM and other adult men in non-criminalized countries — reflecting a range of social and biological factors driving HIV. But in countries with de jure criminalization under law, that difference was 24.8 percentage points and in countries with a de facto policy of enforcement of criminal laws the difference was 30 percentage points.

Behind these trends is, at least in part, the connection between law and the uptake of health services. In criminalized contexts, studies have shown there is increased fear and hiding among gay men and other men who have sex with men, decreased provision and uptake of HIV prevention services, and decreased uptake of HIV care and treatment services.^124^ Rates of ever having taken an HIV test, having taken an HIV test in the past 12 months, and knowledge of HIV status among gay men who live with HIV were 1.25 times, 1.4. times, and 3.2 times higher, respectively, in countries with the least severe anti-LGBTQ+ policies compared to the countries with the most severe anti-LGBTQ+ policies, according to a systematic review of 75 studies from 28 countries.^125^ This evidence bolsters other literature in the space, for example showing the odds of contracting HIV are significantly greater in Caribbean countries that criminalize consensual same-sex sex than in those where such sexuality is legal.^126^ Immediately after the enactment of a harsh antigay law in Nigeria, both avoidance of health care and fear of seeking health care went up significantly.^127^

Moving to a global perspective, there is evidence that avoiding criminalization of consensual same-sex sex benefits the AIDS response not only for LGBTQ+ people but for the entire population. A decade ago the journal *Science* named as “Breakthrough of the Year” new evidence that effective HIV treatment could fully suppress the HIV virus and stop HIV infections.^128^ Studies showed viral suppression not only protects the health of people living with HIV, but also fully stops transmission of HIV to their partners.^129^ At the United Nations, based on this evidence, member states adopted a series of ambitious targets to ensure all people living with HIV know their status and access effective HIV treatment, as a strategy to end the AIDS pandemic by 2030.^130^

In another study using the data from this article, Kavanagh et al. showed in non-criminalizing countries a significantly higher portion of all people living with HIV know their status and a higher portion of all people living with HIV are on effective HIV treatment and successfully suppressed the HIV virus to the point where it is undetectable.^131^ They had 8% higher viral suppression on average and 11% higher knowledge of status when compared to non-criminalizing environments.

The public health impact of laws and policies criminalizing LGBTQ+ people goes beyond HIV. The direct effects and the stigma linked to criminalization have a broad negative effect on mental and physical health.^132^ Studies in Botswana (before decriminalization), Malawi, and Namibia under criminalization found that MSM were twice as likely to be afraid to seek health care and over six times as likely to be refused services than heterosexual people.^133^ A study in Malawi, Mozambique, Namibia, Zambia, and Zimbabwe found that sexual and gender minority adolescents struggle to access sexual and reproductive health care services due to laws criminalizing consensual same-sex sex and social attitudes about adolescent sexuality.^134^ Criminal laws were said to contribute to an environment of state-endorsed homophobia in which health providers were unsure of their responsibilities (e.g., to report those seeking care to police) or felt validated in refusing services, and health seekers were afraid of accessing necessary care. In Malawi, challenges of violence and discrimination in health care facing LGBTQ+ people have been exacerbated by the 2016 suspension of the 2012 moratorium on arrests and prosecutions for consensual same-sex sex.^135^ The passage of anti-transgender legislation in the US was linked to suicide- and depression-related internet searches, and the failure of anti-transgender bills was associated with fewer depression-related searches.^136^ After passage of a bill on gender-affirming care and identity, transgender people in Argentina reported a reduction in avoidance of the health care system due to discrimination — with only 5.3% avoiding health care settings out of fear of discrimination, compared to 41.2% prior to the enactment of the law. After the law passed, 12.7% reported teasing and assault by health care facility personnel, versus 40.2% before the law.^137^

## Legal and Political Pathways to Decriminalization: Recent National Cases

Around the world, many jurisdictions in which decriminalization had once seemed difficult or impossible have recently removed the criminalization of same-sex sexuality. They represent a wide range of different cultural, religious, geographic, and historical context — and in each decriminalization has become possible in the last few years. These contexts have shaped the path to decriminalization and each country’s journey to decriminalization has been quite different.

A few cross-cutting lessons stand out: First, initial efforts often failed — sometimes necessitating a shift of venues between legislative and judicial arenas, sometimes multiple times over the course of years. The shifts at first looked like failures at law reform, but ultimately reinforced each other toward change in these countries. Second, in most of the countries reviewed, important champions for legal change — what political scientists call policy entrepreneurs^138^ — are found in multiple arenas, usually within government in the legislature or executive, in the judiciary, and in civil society. Those champions — sometimes individuals and sometimes organizations — were critical in pushing change. As MacNaughton and Duger show, these actors are likely crucial for the various processes of domestic legal change including internalization of global norms and acculturation over time through persuasion or long-running interaction between domestic and international actors.^139^ And third, they have taken very different paths to reach the same outcome of decriminalization — in many, the efforts were quite public, others were accomplished behind the scenes; in some, strong anti-rights mobilization had to be overcome before law reform could occur while in others the process was more of socialization of elites; and in some, the efforts were relatively rapid while others stretched over a decade.

Recent progress in decriminalization has taken place in countries against a backdrop of colonial legacies of criminalization, as well as a history of religious and cultural justifications.^140^ The following section provides select case studies of how countries pursued decriminalization through legislative or judicial approaches. This section also provides context, where available, on the critical role of civil society organizations in advocating for and contributing to the process of legal reform.

### Case Studies of Judicial Approaches

#### Mauritius

Mauritius enacted Section 250 of its Criminal Code in Mauritius in 1898, during British rule. Section 250 provided the offense of “sodomy,” criminalizing anal sex between consenting male adults in private. Following international case law, including judgements from other former colonies that enacted punitive laws during British rule, the Mauritian Supreme Court recently decriminalized same-sex sex by declaring Section 250 of its Criminal Code unconstitutional.

Support for repealing Section 250 had been building up for decades. In 2007, then Mauritius Attorney-General Jayarama Valayden introduced the Sexual Offenses Bill of 2007 in Parliament to repeal Section 250.^141^ The bill never passed, but the efforts to repeal Section 250 pivoted to the courts. These efforts were led by civil society organizations including the Collectif Arc-En-Ciel (CAEC), Young Queer Alliance (YQA), and Love Honor Cherish Foundation. In late 2023 the Supreme Court of Mauritius decided^142^ two complaints filed one month apart by the Young Queer Alliance and the Love Honor Cherish Foundation^143^ and the president of CAEC.^144^ The 1968 Constitution of Mauritius explicitly names “sex” as a ground protected against discrimination. Citing various decisions from other jurisdictions, the Supreme Court of Mauritius found in 2023 that the word “sex” covers protections against discrimination based on “sexual orientation.”

Pleading against decriminalization, the Mauritian government stated that amending Section 250 had been on its agenda but this “remains a highly sensitive issue in Mauritius in view of the delicate socio-cultural and religious fabric” of their society and an amendment could only be introduced “when the necessary conditions favorable to its adoption in Parliament are present.” Rejecting these arguments, the Supreme Court explained that the country was a secular state and explained that Section 250 was not a result of “domestic democratic will” nor “to reflect any indigenous Mauritius values”^145^ but instead “a course imposed on Mauritius and other colonies by British rule.” The court considered the fact that England had decriminalized same-sex sex but Section 250 remained on the books in Mauritius as a contradiction that needed to be remedied.

#### Botswana

Section 164 of the Penal Code in Botswana, promulgated in 1968 before the country secured its independence from British rule, criminalized “carnal knowledge against the order of nature” and one who “permits any other person to have carnal knowledge of him or her against the order of nature.”^146^ Sections 165 and 167 also criminalized same-sex sex.^147^ While these laws were rarely enforced, they were a structural barrier to providing and receiving HIV and other health services and reinforced systemic discrimination against LGBTQ+ populations. A first attempt to challenge their constitutionality failed in 2003, with the court declaring that “the time has not yet arrived to decriminalize homosexual practices even between consenting adult males in private.”^148^

Despite this loss, civil society continued its efforts to decriminalize same-sex sex in Botswana. A key milestone in this fight came in 2016 when the Botswana Court of Appeal ordered the government to register the civil society organization Lesbians, Gays and Bisexuals of Botswana (LEGABIBO) as a society under the Societies Act to advocate for and affirm the rights of Botswana’s LGBTQ+ population.^149^ That same year Letsweletse Motshidiemang filed a motion in the High Court seeking Sections 164(a)(c), 165, and 167 to be declared unconstitutional.^150^ LEGABIBO was admitted as amicus curiae in 2017 and presented evidence showing that same-sex sex criminalization inhibited LGBTQ+ people from effectively accessing medical treatment.^151^

Two years later, the High Court ruled these criminal offenses unconstitutional because of their inconsistency with Section 3 (equal protection), Section 9 (right to privacy), and Section 15 (non-discrimination) of the Botswana Constitution.^152^ Botswana’s Court of Appeal upheld the ruling with the explicit stance that the offenses “serve only to stigmatize gay men unnecessarily” and “serve only to incentivize law enforcement agents and others to become key-hole peepers and intruders into the private space of citizens.”^153^ After the judgment, the president promised to comply with the decision through the principles of democratic governance and the rule of law.^154^

#### Antigua and Barbuda

Civil society organizations were also critical actors in the efforts that led to decriminalization in Antigua and Barbuda. Sections 12 and 15 of the Sexual Offences Act of 1995 criminalized same-sex sexual conduction with harsh penalties that included up to 15 years in prison.^155^ These offenses were struck down by the Antigua and Barbuda High Court in 2022 following an action brought by members of civil society groups Meeting Emotional and Social Needs Holistically and Women Against Rape. The High Court found that Sections 12 and 15 of the Sexual Offences Act 1995 “offends the right to liberty, protection of the law, freedom of expression, protection of personal privacy and protection from discrimination on the basis of sex.”^156^

### Case studies of Legislative Approaches

#### Angola

Article 71(4) of the Angolan Penal Code of 1886 penalized “vices against nature,”^157^ an offense that could have been interpreted as criminalizing consensual same-sex sex. The new Penal Code passed by the Angolan National Assembly passed in 2019 repealed this section and enacted protections against discrimination on the basis of sexual orientation.^158^ Civil society organizations in Angola played a critical role as a driving force for this legal reform,^159^ including through direct advocacy by organizations like the Associação Íris Angola^160^ and with the implementation of programs aimed at improving sexual and reproductive health outcomes among vulnerable populations.^161^

#### Singapore

Civil society groups such as Pink Dot, Ready4Repeal, and Heckin’ Unicorn played a key role driving efforts toward law reform in Singapore.^162^ Well-attended annual pride events and online movements such as #Ready4Repeal were powerful advocacy platforms used by civil society to increase the socialization and social acceptance of LGBTQ+ communities.^163^ The arrest of Tan Eng Hong for committing an act of “gross indecency” in a public restroom also gave rise to the first legal challenge to the constitutionality of same-sex criminalization in 2010.^164^ A gay couple, Lim Meng Suang and Kenneth Chee Mun-Leon, instigated another constitutional challenge. The High Court, however, ruled that Section 377A of the Penal Code criminalizing “any act of gross indecency between males” did not violate the constitution and the Court of Appeal upheld that decision.

Efforts to repeal Section 377A through the courts continued in Singapore, with limited gains. In 2022 the Court of Appeal once again declined to rule Section 377A as unconstitutional.^165^ However, this ruling referenced a 2018 statement made by the attorney general underscoring that the government stressed that police would not proactively enforce this provision and that prosecution would not be in the public interest in cases where “offenses” under Section 377A are committed between two consenting adults in a private place.^166^ The court considered that this statement generated “substantive legitimate expectations” of non-prosecution and may be an indicator of the more lenient perspectives of lawmakers toward LGBTQ+ communities.^167^

Although the court did not rule Section 377A unconstitutional, the decision signaled a greater acceptance of the LGBTQ+ community and decriminalization. This greater acceptance was confirmed by a unique survey concluded in 2022, assessing the views of the general population on the LGBTQ+ community and the criminalizing provision Section 377A.^168^ The survey, launched by the government’s official feedback unit, REACH, was reported to have garnered far higher numbers of responses than other typical national surveys.^169^ Substantial efforts from local civil society organizations contributed to the promotion of the survey.^170^ Later that year the government announced its decision to repeal the criminalizing provision in a televised speech at the National Day Rally in August 2022.^171^ The Penal Code (Amendment) Act 2022 repealing Section 377A of the Penal Code 1871 was passed in November 2022.^172^

#### Cook Islands

Civil society and government groups, including Te Tiare Association — the Cook Islands’ oldest LGBTQ+ organization — played a crucial role in reforming laws that criminalized consensual same-sex sex. The Crime Act of 1969 prohibited same-sex relations under Sections 154 (indecency between males), 155 (sodomy), and 159 (hosting homosexual activities), punishable by up to 10 years in prison.^173^ In 2013, a Crimes Bill was introduced to remove these discriminatory clauses and was reviewed by a parliamentary Standing Committee in 2017.^174^ That same year, Parliament debated a draft bill to decriminalize same-sex sexual conduct between consenting adults.^175^ The UNDP assisted civil society in creating petitions that highlighted the public health benefits of these reforms.^176^ However, a change in political leadership stalled the bill for two years.

Over the next few years, civil society groups continued to engage with policymakers and traditional leaders to address concerns and correct misconceptions. They launched visibility campaigns to raise awareness and acceptance of LGBTQ+ individuals and conducted polls to assess public opinion, which helped build government support, including from opposition leaders. In 2023, these efforts led Parliament to pass the Crimes (Sexual Offences) Amendment Bill, which repealed the sections criminalizing indecency and sodomy.^177^

## Conclusion

The world has seen a remarkable, generational shift in the global legal landscape for LGBTQ+ sexuality in recent years. Through courts and legislatures, countries throughout the world have removed laws criminalizing LGBTQ+ sexuality. When the AIDS pandemic burst into global consciousness, most of the world’s governments designated same-sex sexuality a crime. The US was just one example where the criminalization of sodomy was only removed by the Supreme Court in 2003. Today, our analysis shows two-thirds of the world’s governments do not criminalize consensual, adult same-sex sex under law — either because they are among the minority of countries that never did or because they decriminalized as Mauritius, Singapore, and many other countries have recently done. Meanwhile, in a significant portion of these countries that do criminalize same-sex sexuality under law, a de facto policy of nonenforcement can be seen — a legal posture that proved the starting point for all of the governments that recently decriminalized. At the same time, there is a clear and coordinated countermovement pushing to deepen criminalization, primarily in a set of countries that currently criminalize. But if one were only paying attention to media outlets from the Global North or rhetoric of some politicians, one could be excused for not understanding the degree to which these are increasingly outliers from a growing normative consensus. Today there is far less a “world divided” on whether LGBTQ+ people should be criminalized for their sexuality than there is a concerned anti-rights movement pushing against this normative growth and, in some countries, succeeding — to the detriment of LGBTQ+ people. As our analysis shows, this is also to the detriment of global public health as countries fail to realize the health benefits of removing criminalizing laws and replacing them with rights’ protection.

In the recent waves of decriminalization, and in those potentially to come, this process has largely meant removing laws imposed during colonialism that then persisted for decades. While anti-rights actors often try to portray decriminalization as a “western” agenda, decisions by judges and legislators in Africa, Asia, and the Caribbean have rightly pointed out that it was the criminalization itself that was imported. As the case studies in this article show, removing those laws has taken significant political energy and the process has varied greatly depending on the particular context. But in case after case, where decriminalization once seemed impossible, it was realized when policy entrepreneurs from government and civil society mobilized through early legal setbacks. Where some see changing the structural determinants of health as too hard or too complex, we argue that these investments not only succeed but they also bring with them concrete benefits.
